# A Specific High-Protein Weight Loss Program Does Not Impair Renal Function in Patients Who Are Overweight/Obese

**DOI:** 10.3390/nu14020384

**Published:** 2022-01-17

**Authors:** Anne-Sophie Truche, Sébastien Bailly, Odile Fabre, Rémy Legrand, Philippe Zaoui

**Affiliations:** 1 AGDUC, Voiron Dialysis Center, 38500 Voiron, France; ahainauttruche@agduc.com; 2 HP2 Laboratory, Grenoble Alpes University, INSERM U1300 and Grenoble Alpes University Hospital, 38000 Grenoble, France; 3 Groupe Éthique et Santé, Actiburo 1, Bâtiment A—100 Chemin de l’Aumône Vieille, 13400 Aubagne, France; odile.fabre@groupethiquetsante.com (O.F.); remy.legrand@groupethiquetsante.com (R.L.); 4 Service de Néphrologie, Dialyse, Transplantation Rénale, Grenoble Alpes University Hospital, 38700 La Tronche, France; pzaoui@chu-grenoble.fr; 5 AGDUC, Meylan Dialysis Center, 38240 Meylan, France

**Keywords:** weight loss, chronic kidney disease, obesity, high protein

## Abstract

Although high-protein diets appear to be the most efficient way to lose weight, concerns may arise about their innocuity on renal function. The objective of this study is to assess the impact of a weight loss program on renal function. A multicentric cohort-based study was performed using the RNPC© French national weight loss program. Patients with at least two creatinine measurements at the beginning of the program and at the end of the weight loss phase between 1 January 2016 and 1 July 2021 were included. Renal function was assessed by Modification of Diet in Renal Disease (MDRD) equation-based estimated glomerular filtration rate (eGFR). From 4394 patients with two creatinine measurements included, 1579 (35.9%) had normal eGFR (MDRD 90–120 mL/min/1.73 m^2^), 210 (4.8%) had hyperfiltration (MDRD > 120 mL/min/1.73 m^2^), 2383 (54.2%) had chronic kidney disease (CKD) grade 2 (MDRD 60–90 mL/min/1.73 m^2^), and 221 (5.0%) had CKD grade 3 (MDRD 30–60 mL/min/1.73 m^2^). Multivariable analyses showed no eGFR change for patients in initial CKD grade 2, normal eGFR and hyperfiltration, and a significant increase in CKD grade 3. The RNPC© program avoids renal function impairment during the two first phases, regardless of the initial eGFR.

## 1. Introduction

Worldwide, 39% of the population is overweight, of which 13% are obese, leading to overweight and obesity being considered as one of the major 21st-century health care challenges [1]. This has been highlighted by the current SARS-CoV-2 pandemic. Being overweight or obese has been associated with deleterious renal effects and a higher risk of chronic kidney disease (CKD), even when the confounding effect of its associated comorbidities (i.e., hypertension and diabetes) is removed [2]. Hence, a new specific entity among renal diseases has emerged, named under the term obesity-related glomerulopathy (ORG) [3]. Weight reduction is one of the objectives of CKD management in the Kidney-Disease Improving Global Outcomes (K-DIGO) recommendations [4] to achieve a healthy body weight (body mass (BMI) 20–25 kg/m^2^). Indeed, in a large English cohort-based retrospective study, at BMI 40 kg/m^2^, a median of 13% intentional weight-loss—by whichever mean to obtain it—reduced the relative risk of type 2 diabetes (T2D) by 41%, sleep apnea by 40%, hypertension by 22%, dyslipidemia by 19%, and asthma by 18% [5]. Furthermore, achieving a 13% intentional weight loss, compared to maintaining a stable BMI, reduced the risk of hypertension and CKD in higher BMI categories (i.e., >35 kg/m^2^) [5].

Several strategies are available to achieve weight loss, i.e., dietary and lifestyle modifications, pharmacological treatments, and bariatric surgery. The first appears as the least expensive option and, despite achieving a less impressive weight loss than bariatric surgery [6], can already provide significant results in improving the health of patients who are obese. A modest diet-induced weight loss (5–10% of body weight) in patients with class I and II obesity (i.e., from 30 to 39.9 kg/m^2^) already decreased left ventricular mass and improved systolic and diastolic function, as well as vascular thickness [7]. Similarly, a significant enhancement of several metabolic parameters associated with cardiovascular risk was noted in T2D patients with such a moderate weight reduction [8]. At least, a meta-analysis including randomized controlled trials (RCTs) with weight-reducing diets found that they were associated with an 18% relative reduction in premature mortality, although no benefit was proven concerning cardiovascular mortality or new events [9].

In the DiOGenes study, a multicenter RCT compared five different diets, which were a combination of low- or high-macronutrient (proteins, high or low-glycemic-index carbohydrates and lipids) proportions or a control diet, for their ability to ensure obese families maintain their weight loss after an initial phase of 8% weight reduction. Patients assigned to a high-protein diet with low-glycemic-index carbohydrates were more likely to maintain their weight loss after six months. Moreover, high-protein diets led to a further decrease in patients’ weight than low-protein diets [10]. However, concerns may arise on their risks of impairing renal function [11], especially for patients with comorbidities linked to a higher risk of kidney impairment or already suffering from overt CKD.

RNPC© (for Rééducation Nutritionnelle et Psycho-Comportementale, i.e., the Nutritional Psycho-Behavioral Reeducation) program, based on a high-protein, low-carbohydrate, and low-fat diet, has already proven to be an efficient [6] and cost-effective [12] weight loss intervention with significant improvement in metabolic parameters following its weight loss phase [13]. The main objective of this study was to assess whether the RNPC© program is associated with a modification of patients’ Modification of Diet in Renal Disease (MDRD) equation-based estimated glomerular filtration rate (eGFR) during the program high-protein phases, i.e., weight loss and early stabilization phases.

## 2. Materials and Methods

### 2.1. RNPC© Program

This was a retrospective study from the French RNPC© program, which has already been described elsewhere [12]. Briefly, RNPC© is a three-phase dietary program carried out in 76 French centers, its specificity being a special emphasis on long-term maintenance of the achieved weight loss thanks to a close follow-up by dieticians (twice a month). The RNPC© diet is composed of two meals per day, including vegetables (ad libitum) and one fruit (among a list of low-calorie and low-glycemic-index vegetables and fruits provided to the patients by the dietician), animal proteins (from meat, fish, eggs, or shellfish; 100–200 g/meal), and commercially available meal supplements (these do not replace a meal), in the number of four units for men and three for women, in the form of snacks (biscuits, cereal bars, bread, crackers, soups, omelets, drinks, and desserts), ready to eat or easy to prepare, for which calorie content does not exceed 200 kcal/unit and which the patients can eat whenever they want during the day.

The contribution of ad libitum vegetables to the total calorie intake is considered negligible, and as their glycemic index is low due to their richness in fibers, they rapidly induce satiety for a moderate quantity.

The RNPC© meal supplements were selected to support a high level of high-quality proteins (containing an average of 110 kcal with a maximum of 200 kcal, 15.8 g proteins, 2.4 g carbohydrates, 5.4 g fats, and 2.3 g fiber, for an average serving size of 30 g), and these were fortified with vitamins and minerals to avoid deficiencies caused by a low energy intake.

During the weight loss phase, the RNPC© meal supplements contributed approximately 40% of the total energy intake, and daily protein intake corresponded to 60% of total energy (i.e., 1.5 g/kg in men and 1.2 g/kg in women), with an adaptation in case of CKD (0.8 to 1 g/kg in case of CKD grade 3 and ≤0.6 g/kg in case of CKD grade 4), as recommended by French health authorities (Haute Autorité de Santé). The second part of the program was a stabilization phase, during which energy intake was gradually increased in five steps. Between each of the five steps of the stabilization phase, energy intake increased according to the calculation: next energy level = previous energy level + (post-weight loss energy requirement—weight loss energy intake)/5. Post-weight loss energy requirement was determined by Black’s formula [14] using a sedentary physical activity level of 1.375. During this phase, the number of meal supplements, as well as percentage of dietary protein in total energy intake, were progressively decreased by suppressing one high-protein meal supplement per step of the stabilization phase, targeting a macronutrient composition of 25% proteins, 45% carbohydrates, and 30% fats at the transition to the weight maintenance phase, i.e., completion of the program. The third phase was the maintenance phase, where energy intake matched the post-weight loss energy requirement—thereby, energy balance was achieved, during which dietary consultations can be continued on an optional basis. Patients with BMI > 25 kg/m^2^ or a high waist circumference (WC) (≥94 cm for men and ≥80 cm for women) with or without associated comorbidity can join the program provided they obtain the authorization of their physician. A biological checkup with creatinine measurement was needed before entering the program to rule out an underlying CKD, and physicians were advised to renew it during the program.

The duration of each phase was variable, depending on how quickly patients reached their personal objective of weight. According to Thorning et al. [12], the median duration of the weight loss phase was 111 days (IQR: 57; 182) in women and 92 days (IQR: 48; 157) in men. The median for the full program was 258 days (IQR: 196; 362) for women and 230 days (IQR: 167; 318) for men.

### 2.2. Data Definitions

CKD was defined according to K-DIGO guidelines [4]. Delta MDRD was calculated as the difference between final and initial MDRD measures.

Bodyweight, fat mass, and fat-free mass were measured at baseline and after each visit by the dieticians using a calibrated bioelectrical impedance scale (Beurer BG42, Ulm, Germany). Height was measured to the nearest centimeter at baseline using a height gauge. BMI was obtained using the formula: body weight [kg]/(height [m])^2^. WC was measured to the nearest centimeter at the natural waist. However, for patients with abdominal adiposity with no visible natural waist, the measurement was taken at the level midway between the lowest rib and the iliac crest, approximately 2–5 cm above the navel. Patients with hypertension were detected based on their antihypertensive medication. Patients with diabetes were detected based on their initial glycemia (superior or equal to 1.26 g/L) and/or antidiabetic medication.

### 2.3. Population

Patients with an initial MDRD equation-based eGFR > 30 mL/min/1.73 m^2^ who participated in the RNPC© program between 1 January 2016 and 1 July 2021, were included if they had a second serum creatinine measurement, either at the end of the weight loss phase or during the stabilization phase. Exclusion criteria were pregnancy, breastfeeding, and patients less than 18 years of age. Data of patients entering into the program were registered in a database authorized by the French Data Protection Authority (CNIL).

### 2.4. Outcomes

Our main outcome was eGFR evolution. Different subsets of patients based on their renal function at baseline and their comorbidities (hypertension, diabetes, or none) were explored.

### 2.5. Statistical Analysis

Quantitative variables were summarized as median (interquartile range) and qualitative variable with frequency and percentage. Comparisons between groups were performed by using the chi-square test for qualitative variables or Kruskal–Wallis test for quantitative variables. Finally, a linear mixed model adjusted on age and sex with random patient effect was used to assess the evolution over time for MDRD and anthropometric parameters according to the initial MDRD class. Sensitivity analyses were performed for (1) comorbidities sub-groups (hypertension, diabetes), (2) sub-group of patients without hypertension or diabetes, and (3) patients in the weight loss phase of the RNPC© program for the two measures. Finally, a linear multivariable regression was performed to identify factors associated with delta MDRD in which the dependent variable was the delta MDRD (final value—initial value), and the variable of interest was a time in months from initial measures. A point plot with a confidence interval is provided to show the effect size for all patients and for each initial MDRD class. Confounders were: initial MDRD, initial BMI, age, sex, diabetes, hypertension, and time between two measures. Correlations between variables introduced in the model were checked to avoid the introduction of collinear variables, and the normality of residuals was assessed to verify the model assumption. Statistical analyses were performed with SAS v9.4 (SAS Institute, Inc., Cary, NC, USA). A *p*-value threshold of 0.05 was considered significant.

## 3. Results

### 3.1. Population

A total of 4394 patients had two creatinine measurements and were included in the present study. The median MDRD was 85 mL/min/1.73 m^2^ (74; 97) and patients were divided as follows: 1579 (35.9%) had normal eGFR (MDRD 90–120 mL/min/1.73 m^2^), 210 (4.8%) had hyperfiltration (MDRD > 120 mL/min/1.73 m^2^), 2383 (54.2%) had CKD grade 2 (MDRD 60–90 mL/min/1.73 m^2^), and 221 (5.0%) CKD grade 3 (MDRD 30–60 mL/min/1.73 m^2^). The median age was 57 years old (48; 65), with a majority of women (3379 (76.9%)). Considering patients with information about medical prescription (*N* = 3283, 74%), 504 patients were diabetic (15.4%), 1731 had hypertension (52.8%), and 1383 (31.5%) had no hypertension and no diabetes. There were significant differences between groups for initial anthropometric values (Table 1).

Patients in CKD grade 3 (MDRD 30–60 mL/min/1.73 m^2^) were significantly older, had more comorbidities (hypertension and/or diabetes) and had an initial weight and BMI higher than patients in other MDRD classes.

### 3.2. Evolution of the Anthropometric Measures

Between the two creatinine measurements, with a median duration between two blood samples of 238 days (205; 282), patients significantly reduced their weight (delta weight by 14.5 kg (19.9; 10.1), *p*-value < 0.01), which corresponded to a decrease of 5.3 kg/m^2^ (7.2; 3.7) of BMI, and there was a significant difference according to initial MDRD classes, with patients in CKD grade 3 having a lower weight reduction. Over time, there was a significant increase in muscle mass in each group (delta muscle mass for all patients final measure-initial measure: 1.3% (0.5; 2.1), *p*-value < 0.01), and there was a slightly significant difference in increase according to MDRD classes, with a lower increase for CKD grades 2 and 3 (1.2% (0.4; 2.1) and 1.2% (0.5; 2.1), respectively) (Table 2).

By comparing patients in CKD grade 3 to other categories, the evolution of weight, BMI, and fat mass were significantly lower for these patients.

### 3.3. Evolution of the MDRD Measures

**Figure 1:**
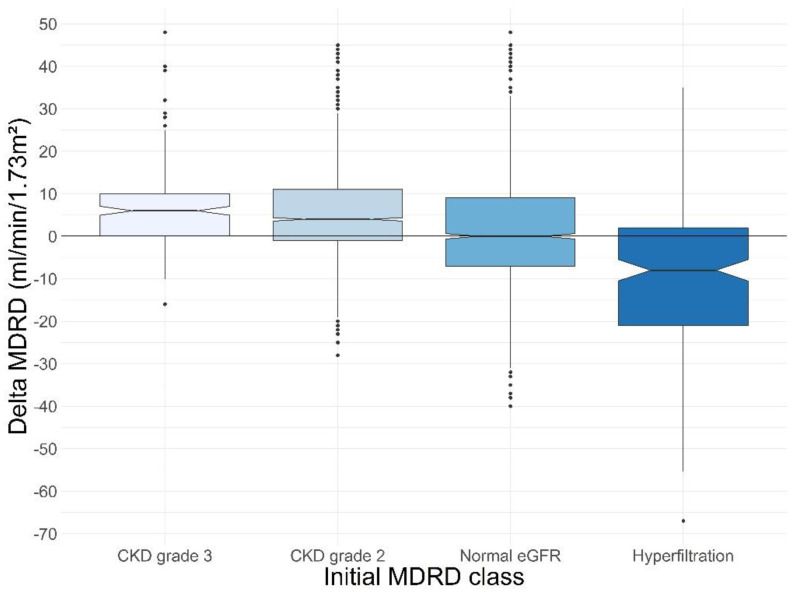
Distribution of delta MDRD according to initial MDRD class.

Patients significantly increased their eGFR (85 (74; 97) mL/min/1.73 m^2^ vs. 88 (77; 101) mL/min/1.73 m^2^, *p*-value < 0.01), resulting in an overall increase of 3 mL/min/1.73 m^2^ (−3; 10). Similar results were found for patients with a normal initial eGFR (98 (93; 106) mL/min/1.73 m^2^ vs. 100 (94; 108) mL/min/1.73 m^2^, *p*-value < 0.01), as well as patients with CKD grade 2 (78 (71; 83) vs. 79 (72; 85) mL/min/1.73 m^2^, *p*-value < 0.01) and patients with CKD grade 3 (54 (49; 57.5) vs. 59 (52; 65) mL/min/1.73 m^2^, *p*-value < 0.01), who experienced an increase in MDRD values (Table 3 and Figure 1). Inversely, patients with hyperfiltration prior to RNPC© program experienced a decrease in their eGFR (130 (124; 138) vs. 123 (109; 137) mL/min/1.73 m^2^, *p*-value < 0.01) (Table 3).

By comparing patients in CKD grade 3 to other MDRD classes, there was a significant increase in MDRD of 6 mL/min/1.73 m^2^ (0; 10) vs. 2 (−4; 10) for the three other classes combined (*p* < 0.01).

**Figure 2:**
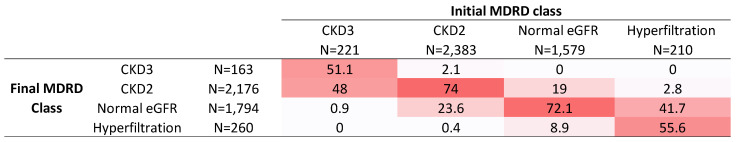
Changes in MDRD classes over time (%).

**Figure 3:**
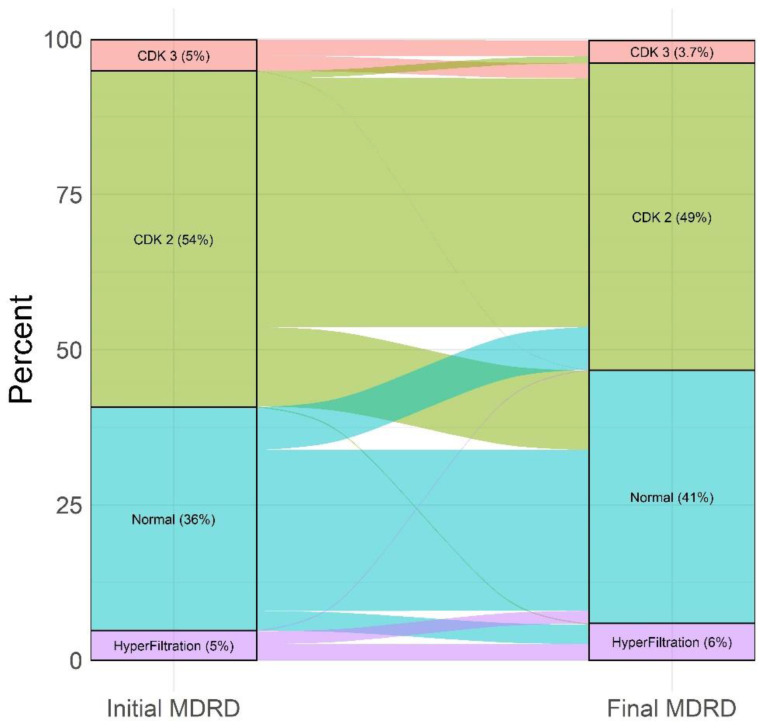
Evolution of MDRD classes over time (%).

Overall, there were significant changes in the proportion of MDRD classes over time. From the 221 patients initially in CKD grade 3, 106 (48%) moved to CKD grade 2 and 2 (1%) to normal eGFR. From the 2383 patients in CKD grade 2, 562 (23.6%) move to normal eGFR. Conversely, 50 (2.1%) and 8 (0.4) moved to CKD grade 3 or hyperfiltration, respectively. From patients with normal eGFR, 301 (19%) moved to CKD grade 2 and 138 (8.7%) to hyperfiltration. Finally, from 210 patients with hyperfiltration, 90 (42.9%) moved to normal eGFR and 6 (2.8%) to CKD grade 2 (Figure 2 and Figure 3). Figure 2 shows that, for each initial MDRD class, most of the patients remained in the same MDRD class.

### 3.4. Sensitivity Analyses

eGFR was significantly increased during the program for patients with diabetes (86 (71; 100) vs. 87 (74; 103) mL/min/1.73 m^2^, *p*-value < 0.01) or hypertension (82 (70; 94) vs. 85 (74; 98) mL/min/1.73 m^2^, *p*-value < 0.01). GFR also increased for normotensive and non-diabetic patients (85 (76; 97) vs. 89 (78; 101) mL/min/1.73 m^2^, *p*-value < 0.01). By considering patients with two measures only in the weight loss phase of the program, the change was similar to overall results (86 (74; 98) vs. 88 (77; 100) mL/min/1.73 m^2^, *p*-value < 0.01) (Table 4).

### 3.5. Multivariable Analysis

**Figure 4:**
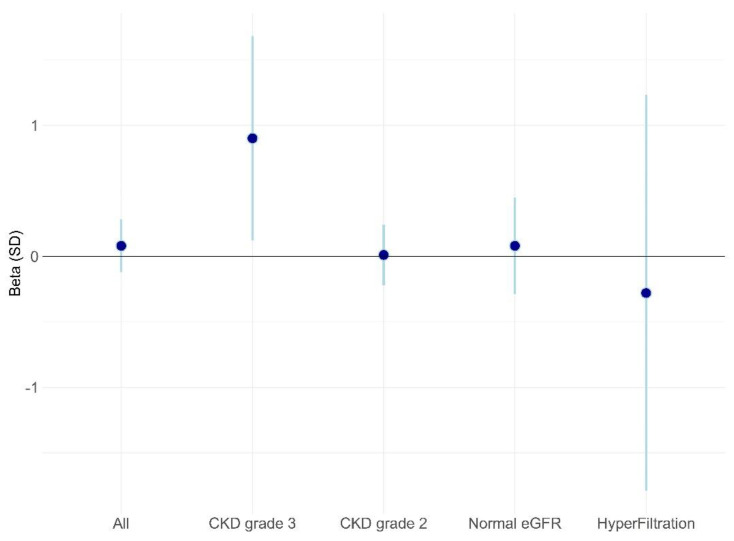
Effect of time on delta MDRD from multivariable linear model according to MDRD classes. Beta(SD): Effect size and 95% confidence interval for time effect on change in MDRD (final value—initial value) according to the initial class of MDRD from multivariable linear regression adjusted by age, sex, initial MDRD, and initial BMI.

Using multivariable linear regression, the main factors associated with a decrease in MDRD at the final measure were the initial MDRD value (beta = −0.19 (standard deviation (SD): 0.01), *p* < 0.01), sex (women) (beta = −1.03 (± 0.46), *p* = 0.03), and history of diabetes (beta = −1.69 (±0.56), *p* < 0.01). The initial BMI value was associated with an increase in the MDRD value (beta = 0.08 (±0.04), *p* = 0.03). The duration between two measures, age, or history of hypertension were not associated with a change in MDRD over time (*p* = 0.37, 0.15, and 0.54, respectively), but were considered confounding factors in the final model. By considering each eGFR class, there was only a significant increase in delta MDRD by month for patients in initial CKD3 (Beta: 0.90 (0.12; 1.68), *p* = 0.02), i.e., for each month of RNPC© program, there was an increase of 0.90 mL/min/1.73 m^2^ of MDRD. Conversely, there were no significant changes in delta MDRD for patients in CKD2 (Beta = 0.01 (−0.22; 0.24), *p* = 0.96), normal eGFR (Beta = 0.08 (−0.29; 0.45), *p* = 0.66), or hyperfiltration (Beta = −0.28 (−1.79; 1.23), *p* = 0.72) classes (Figure 4).

## 4. Discussion

In our study, a moderate high-protein, low carbohydrate diet, i.e., the RNPC© program, was followed by patients who are overweight or obese, of whom roughly half suffered from hypertension and 15% suffered from diabetes, known CKD risk factors. This program has been proved to be efficient in reducing patients’ weight by a median of 14.5 kg (19.9; 10.1) and improving their anthropometric parameters. Meanwhile, a global increase in estimated renal function during the two first phases of the program was noted in patients with normal eGFR, CKD grades 2 and 3, whereas eGFR decreased in patients with hyperfiltration. This effect was maintained in the different subsets of patients suffering from comorbidities, as well as in patients without hypertension or diabetes. In multivariable analysis, after adjustment for confounding factors, we showed only a significant change of MDRD by months for patients with CKD grade 3 but not for patients in CKD grade 2, normal eGFR, or hyperfiltration, for whom no significant change can be considered.

Obesity is responsible for hyperfiltration, glomerulomegaly, and adaptative focal glomerulosclerosis secondary to podocyte failure to cover the larger surface area despite hypertrophy [15], resulting in proteinuria and subsequently CKD [16]. Interventions aiming at reducing patients’ weight thus appear to be a reasonable objective to avoid a dramatic increase in CKD incidence linked to ORG and other comorbidities-associated nephropathies.

Bariatric surgery is the most effective option for reducing weight and subsequently maintaining weight loss [17]. It can reverse some of the burdens linked to obesity, such as sleep apnea, T2D, dyslipidemia, and hypertension [17]. Thus, bariatric surgery seems to result in favorable renal outcomes by decreasing creatinine and albuminuria [18], as well as cardiovascular outcomes [19]. However, the surgical option can lead to complications, the prevalence increasing with the severity of CKD [20].

Two therapeutic drug classes that result in moderate weight reduction have emerged these last years, namely glucagon-like receptor peptide 1 receptor agonists (GLP-1RAs) and sodium-glucose cotransporter-2 inhibitors (SGLT2-i). Both have proven to be associated with favorable cardiovascular outcomes [21,22,23]. GLP-1RAs also appear to be renoprotective, mostly driven by a reduction in new-onset macroalbuminuria [21,24], but to date, no study with renal function as the main outcome is available yet. However, the effect of these therapeutics is mediated not only by weight reduction but also through their glucose-lowering effect, reduced blood pressure, and maybe by direct effects on the kidney [25]. Regarding SGLT2-i, the CREDENCE trial was specifically designed to assess directly renal outcomes with a composite criterion of end-stage renal disease, doubling of creatinine level, and death from renal causes, which was decreased by 34% with canagliflozin use in patients with T2D [26]. The DAPA-CKD trial also offered promising results for patients with CKD and proteinuria, even in the absence of diabetes [27]. Similarly, the SGLT2-i renal-protective effect is not limited to its weight effect [27,28]. It is thus difficult to infer a conclusion from these pharmacological intervention studies for the specific effect of moderate weight reduction on renal function and other outcomes.

The RNPC© program positions itself as an alternative to bariatric surgery [6] or before pharmacological options, as a first step not only for patients with simple overweight or grade I or II obesity but also for patients already suffering from obesity-linked comorbidities, such as T2D, hypertension, fatty liver disease, sleep apnea, or arthrosis, or before considering a surgical option. It also has to be seen as a useful complement to other therapeutic options. Large RCTs examining the renal effects of a non-surgical and non-pharmacological option for weight loss are still missing [3], but consistent data are in favor of a significant antiproteinuric effect of a decreased weight [29,30]. In our study, changes in eGFR were also observed in patients without hypertension or diabetes, indicating that the effect of weight loss on renal function was not only mediated by better control of obesity-associated comorbidities. Indeed, other injury pathways have been implicated, notably a role of increased inflammation with adipokine dysregulation, neurohumoral activation, and increased sodium reabsorption [31]. Similar to our study, a recent RCT trial assessing the effect of a weight-loss lifestyle intervention based on an energy-restricted Mediterranean diet showed favorable one-year outcomes by reducing eGFR decline [32]. However, the mean achieved weight loss was only 3.7 kg, which seems lower than the 8% objective of weight reduction of the study and much lower than the magnitude of weight reduction obtained in our study.

Indeed, high-protein, low-carbohydrate diets have largely gained popularity in recent years due to their higher efficiency in weight reduction [10]. However, from a nephrological point of view, when considering a diet based on increased protein consumption, physicians may be reluctant to its application, even in patients without CKD. A high protein load is suspected to induce glomerular hyperfiltration and to promote de novo CKD or accelerate CKD progression, notably by increasing proteinuria, as well as electrolytic and acid–base disturbances [11].

Therefore, a clear dilemma appears: should we advise these high-protein diets to our patients, given the benefits associated with weight reduction in this high-risk population? Conflicting results concerning renal outcomes with such diets can be found in existing literature [33]. As an example, in a cohort study based on women enrolled in the Nurses’ Health Study, a high-protein intake was not associated with adverse renal effects in women with normal GFR at baseline [34], whereas two recent prospective studies suggest that a high-protein diet could result in a faster decline of GFR [35,36]. Nevertheless, these studies were rather based on patients’ dietary habits, and thus on long-term high-protein diet exposition, contrary to our study, where only a transitory increase in protein consumption was considered (weight loss phase median duration: 148 (99–224) days). Moreover, in an RCT testing a low-fat versus a high-protein, low-carbohydrate diet, Friedman et al. showed that a high-protein, low-carbohydrate diet was associated with no harmful effect on kidney function in patients who are obese without pre-existing kidney disease or associated comorbidities, even with 2 years of application [37]. Similarly, in our study, the diet modification was not associated with a deleterious renal effect, even in at-risk patients with pre-existing diabetes or hypertension.

It should be emphasized that the high-protein label of the RNPC© program is mainly based on the protein proportion in the total energy intake in the weight loss phase—i.e., 60%. In fact, the RNPC© program could be considered a moderately high-protein diet because it is limited to 1.5 g/kg/d in men and 1.2 g/kg/d in women. Moreover, in a patient with overt CKD, despite the weight loss phase being a time-limited phase, special attention was given to reducing the absolute protein intake according to national guidelines. Thus, patients with initial eGFR ranging from CKD grade 2 to hyperfiltration were the population truly exposed to an absolute and relatively high-protein diet. In this specific population, no adverse event on renal function was noted. Similarly, in patients with overt CKD exposed to a relatively high-protein diet, the MDRD value increased, which indicates that the RNPC© program is safe in this subset of patients and could help them to stabilize or even moderately improve their eGFR thanks to the diet’s weight-loss-induced beneficial effect. Whether the high-protein load-induced dilatation of the afferent arteriole and increased intraglomerular pressure, which could both explain the observed improvement of renal function, could not be ruled out [11]. However, no worsening of the hyperfiltration state was observed in patients with eGFR > 120 mL/min/1.73m^2^, and eGFR improvement was noted even in patients with overt CKD who had a 0.8 to 1 g/kg/day protein intake.

The median delta MDRD in our study was 3 mL/min/1.73 m^2^, this improvement being statistically significant. However, one should be cautious when interpreting this result and whether this result is of clinical significance remains uncertain. Actually, there is a large discrepancy between eGFR and measured GFR, whichever equation is used, which could result in a patient’s misclassification, and eGFR could also be inaccurate in monitoring renal function over time [38]. eGFR could be performed particularly poorly in some patients’ subsets, such as adolescents [39] or patients with T2D [40]. The best option to estimate GFR in patients who are obese remains an unsolved issue, with specific questions, such as the indexation to body surface area, and even cystatine-C appears to be influenced by body fat [41]. Hence, as an example, in the study by Diaz-Lopez et al. [32], the mean eGFR difference between both groups was very low; meanwhile, no difference in mean urine albumin-to-creatinine ratio was noted, which could testify that the mean eGFR difference did not represent a significant clinical effect of the intervention. Thus far, there is no validated equation for the estimation of the renal function of patients who are obese, and the MDRD equation can be biased in this population [42]. Measured GFR is advised in patient subsets where eGFR could be biased, but could not be performed in the context of our program due to the burden linked to these measures. Our results should, therefore, better be interpreted as an absence of proof of the adverse renal effect of the RNPC© program rather than an improvement of patients’ eGFR.

Several limitations in our study should be acknowledged. First, no albuminuria or proteinuria data were available, and it could be argued that the modification of body composition induced by weight loss could by itself explain the change of a creatinine-based estimation of renal function due to a reduction in muscle mass [42]. Cystatine-C appears to be more reliable in the context of a high-protein diet [43]. Further studies which compare improvement and worsening in eGFR based on such measures can be useful to assess patients who most benefit from the RNPC© program. Moreover, there are no measures for microalbuminuria and urinary creatinine level, which can improve renal function evaluation. In fact, in France, these measures are not commonly prescribed by clinicians and are not systematically reimbursed by health insurance, which explains their absence in this study. Second, the underlying etiology of patients’ nephropathy was unknown. Third, no long-term (i.e., greater than 2 years) evaluation of the patients’ renal function was available. Fourth, bioelectrical impedance is an easy-to-use technique to assess body composition at a low cost; however, this method seems less accurate when body fat is high, which can result in an overestimation of fat-free mass and an underestimation of fat mass [44]. Fifth, the time interval between two serum creatinine measures is not standardized for all patients. However, only measures collected in the high-protein phases, i.e., weight loss and early stabilization phases, were considered for this study, and this time interval was considered a confounding factor in statistical analyses. This variable was not significantly associated with a decreased MDRD over time, which was not in favor of an influence of the duration before renewing creatinine measurement on the results. Lastly, the observational nature of the analysis prevents us from drawing definite conclusions due to confounding factors or the absence of external strict control of patients’ diet, and the results should be confirmed by a randomized controlled trial.

## 5. Conclusions

To conclude, the RNPC© program appears to be a renal-safe weight loss program. This was particularly true in patients without CKD despite its absolutely moderate high-protein compounds. In patients with CKD grade 3 who had an adaptation of their total protein intake, the results were similar, as well as in patients with T2D or hypertension.

## Figures and Tables

**Table 1:** Characteristics of the patients classified according to their MDRD class.

	All (*N* = 4394)	CKD Grade 3 *N* = 221 (5%)	CKD Grade 2 *N* = 2383 (54.2%)	Normal eGFR *N* = 1579 (35.9%)	Hyperfiltration *N* = 210 (4.8%)	Overall *p*-Value
Age	57 (48; 65)	68 (61; 74)	60 (51; 67)	54 (46; 62)	48 (33; 58)	<0.01
Sex (Woman) *N* (%)	3379 (76.9)	146 (66.1)	1871 (78.5)	1208 (76.5)	154 (73.3)	<0.01
Hypertension *N* (%)	1731 (52.8)	148 (72.5)	993 (54.2)	518 (47.3)	71 (49.7)	<0.01
Diabetes *N* (%)	504 (15.4)	48 (23.5)	244 (13.3)	174 (15.90)	37 (25.9)	<0.01
No hypertension No diabetes *N* (%)	1383 (31.5)	50 (22.6)	761 (31.9)	515 (32.6)	57 (27.1)	0.01
**Initial values**						
BMI (kg/m^2^)	33.8 (30.6; 37.7)	34.4 (31.3; 38.1)	33.5 (30.4; 37.4)	33.9 (30.7; 37.8)	35.4 (31.0; 38.9)	<0.01
Weight (kg)	92.2 (81.2; 105.2)	96.9 (81.4; 109.6)	91.3 (80.8; 104.1)	92.5 (81.5; 106.1)	94.9 (83.1; 109.6)	<0.01
Waist circumference (cm)	110 (101; 120)	114 (104; 123)	109 (101; 118)	110 (101; 120)	112 (103; 123)	<0.01
Muscle mass (%)	28.9 (27.0; 31.9)	28.0 (25.6; 31.0)	28.5 (26.8; 31.5)	29.3 (27.5; 32.3)	30.5 (28.0; 33.1)	<0.01
Fat mass (%)	42.0 (38.6; 45.3)	42.7 (39.4; 46.6)	42.1 (38.8; 45.3)	41.6 (38.2; 45.2)	41.5 (37.9; 45.0)	<0.01
Serum creatinine (mg/L)	7.91 (7.0; 9.1)	11.5 (10.4; 13.7)	8.4 (7.8; 9.5)	7.0 (6.5; 7.6)	5.8 (5.3; 6.6)	<0.01
MDRD (mL/min/1.73 m^2^)	85.0 (74.0; 97.0)	54.0 (49.0; 57.5)	78.0 (71.0; 83.0)	98.0 (93.0; 106.0)	130.0 (124.0; 137.5)	<0.01
Duration between two measures (days)	238 (205; 282)	234 (210; 276)	237 (205; 281)	239 (205; 284)	236 (205; 282)	0.89

Values are presented in median (interquartile range) or *N* (%). Hypertension and diabetes: values presented for 3782 patients who had information about medical prescription. BMI: *N* missing = 29; waist circumference: *N* missing = 2; muscle mass: *N* missing = 136; fat mass: *N* missing = 133. CKD: chronic kidney disease, eGFR: estimated glomerular filtration rate; BMI: body mass index, MDRD: Modification of Diet in Renal Disease. *p*-value: chi-square test for qualitative variables and Kruskal–Wallis test for quantitative variables.

**Table 2:** Evolution of anthropometric characteristics over time (final measure—initial measure).

	All (*N* = 4394)	CKD Grade 3 *N* = 221 (5%)	CKD Grade 2 *N* = 2383 (54.2%)	Normal eGFR *N* = 1579 (35.9%)	Hyperfiltration *N* = 210 (4.8%)	Overall *p*-Value
Delta weight (kg)	−14.5 (−19.9; −10.1)	−13.3 (−19.5; −9.4)	−14.3 (−19.4; −10)	−14.8 (−20.7; −10.4)	−14.8 (−21.5; −11.4)	<0.01
Delta BMI (kg/m^2^)	−5.3 (−7.2; −3.7)	−4.8 (−6.7; −3.4)	−5.3 (−7.1; −3.7)	−5.5 (−7.5; −3.8)	−5.4 (−7.7; −4.3)	<0.01
Delta waist circumference (cm)	−16 (−20; −12)	−15 (−19; −12)	−15 (−20; −11)	−16 (−21; −12)	−16 (−21; −12)	<0.01
Delta muscle mass (%)	1.3 (0.5; 2.1)	1.2 (0.4; 2.1)	1.2 (0.5; 2.1)	1.3 (0.6; 2.2)	1.5 (0.6; 2.4)	0.03
Delta fat mass (%)	−4.4 (−6.5; −2.8)	−3.7 (−6.1; −2.1)	−4.3 (−6.4; −2.7)	−4.6 (−6.6; −2.9)	−4.7 (−6.7; −2.7)	0.04

Values are presented in median (interquartile range). Missing values: waist circumference: *N* = 2; muscle mass: *N* = 232; fat mass: *N* = 229. CKD: chronic kidney disease, eGFR: estimated glomerular filtration rate; BMI: body mass index *p*-value: mixed model adjusted on age, sex, and MDRD class.

**Table 3:** Evolution of MDRD measures over time (final measure—initial measure).

	All (*N* = 4394)	CKD Grade 3 *N* = 221 (5%)	CKD Grade 2 *N* = 2383 (54.2%)	Normal eGFR *N* = 1579 (35.9%)	Hyperfiltration *N* = 210 (4.8%)	Overall *p*-Value
Initial MDRD	85 (74; 97)	54 (49; 58)	78 (71; 83)	98 (93; 106)	130 (124; 138)	<0.01 *
Final MDRD	88 (77; 101)	59 (52; 65)	81 (74; 89)	100 (92; 110)	122 (109; 136)	<0.01 *
Delta MDRD	3 (−3; 10)	6 (0; 10)	4 (−1; 11)	0 (−7; 9)	−8 (−21; 2)	<0.01 *

Values are presented in median (interquartile range) or *N* (%). Missing values: waist circumference: *N* = 2; muscle mass: *N* = 232; fat mass: *N* = 229. CKD, chronic kidney disease, MDRD: Modification of Diet in Renal Disease. *p*-value: * Kruskal–Wallis test for quantitative variables.

**Table 4:** Sensitivity analyses.

	All	CKD Grade 3	CKD Grade 2	Normal eGFR	Hyperfiltration	Overall *p*-Value
Diabetes *N* (%)	503 (100)	48 (9.5)	244 (48.5)	174 (34.6)	37 (7.4)	
Initial MDRD	86 (71; 100)	52 (46.5; 55)	77 (70; 83)	100 (94; 108)	132 (126; 136)	<0.01
Final MDRD	87 (74; 103)	55 (46.5; 62.5)	79 (72; 89)	102 (89; 113)	119 (110; 138)	<0.01
Delta MDRD	1 (−5; 10)	2.5 (−1.5; 8.5)	3 (−4; 10)	0 (−10; 10)	−8 (−22; 5)	<0.01
Hypertension *N* (%)	1730 (100)	148 (8.5)	993 (57.3)	518 (29.9)	71 (4.1)	
Initial MDRD	82 (70; 94)	53 (48; 57)	76 (69; 82.8)	97 (93; 105)	131 (124; 137)	<0.01
Final MDRD	85 (74; 98)	57.5 (50; 64.5)	80 (73; 88)	101 (92; 110)	125 (110; 138)	<0.01
Delta MDRD	3 (−3; 10)	5 (0; 9)	4 (−2; 11)	0 (−6; 11)	−3 (−21; 3)	<0.01
No diabetes and No hypertension *N* (%)	1383 (100)	50 (3.6)	761 (55)	515 (37.2)	57 (4.1)	
Initial MDRD	85 (76; 97)	56 (53; 58)	78 (72; 83)	98 (93; 106)	129 (123; 137)	<0.01
Final MDRD	89 (78; 101)	63 (55; 67)	82 (74; 89)	100 (92; 110)	118 (108; 132)	<0.01
Delta MDRD	3 (−4; 10)	8.5 (1; 14)	4 (−1; 11)	0 (−7; 9)	−10 (−19; −1)	<0.01
Weight loss period *N* (%)	1243 (100)	77 (6.2)	647 (52)	451 (36.2)	70 (5.6)	
Initial MDRD	86 (74; 98)	55 (51; 58)	78 (70; 83)	99 (94; 107)	130 (123; 136)	<0.01
Final MDRD	88 (77; 100)	59 (52; 65)	81 (73; 89)	99 (91; 109)	124 (114; 141)	<0.01
Delta MDRD	2 (−4; 10)	5 (−1; 9)	4 (−2; 10)	0 (−8; 9)	−5 (−18; 4)	<0.01

Values are presented in median (interquartile range) or *N* (%). CKD, chronic kidney disease, eGFR: estimated glomerular filtration rate, MDRD: Modification of Diet in Renal Disease. *P* value: Kruskal–Wallis tests to compare four groups.

## Data Availability

Data are available from corresponding authors upon request.
